# Preparation and Properties of Micron Near-Spherical Alumina Powders from Hydratable Alumina with Ammonium Fluoroborate

**DOI:** 10.3390/ma18194589

**Published:** 2025-10-02

**Authors:** Yi Wei, Jie Xu, Jie Jiang, Tairong Lu, Zuohua Liu

**Affiliations:** 1 College of Chemistry and Chemical Engineering, Chongqing University, Chongqing 401331, China; liuzuohua@cqu.edu.cn; 2 Institute of New Functional Materials, Guangxi Institute of Industrial Technology, Nanning 530200, China; gxkjltr188@aliyun.com; 3 State Key Laboratory of Solidification Processing, MIIT Key Laboratory of Radiation Detection Materials and Devices, School of Materials Science and Engineering, Northwestern Polytechnical University, Xi’an 710072, China; 4 Shaanxi Shuimu Xusheng New Material Technology Co., Ltd., Xi’an 710116, China; 5 Guangxi Key Laboratory of Information Materials, School of Materials Science and Engineering, Guilin University of Electronic Technology, Engineering Research Center of Electronic Information Materials and Devices, Ministry of Education, Guilin 541004, China; 19833187050@163.com

**Keywords:** alumina powders, hydratable alumina, ammonium fluoroborate, morphology, thermal conductivity

## Abstract

Micron-sized near-spherical α-Al_2_O_3_ powders are widely used as thermal fillers due to their high thermal conductivity, high packing density, good flowability, and low cost. During the high-temperature calcination, the resulting α-Al_2_O_3_ powders often exhibit an aggregated worm-like morphology owing to limitations in solid-state mass transfer. Researchers have employed various mineralizers to regulate the morphology of α-Al_2_O_3_ powders; however, the preparation of micron-sized highly spherical α-Al_2_O_3_ powders via solid-state calcination is still a great challenge. In this work, micron-sized near-spherical α-Al_2_O_3_ powders were synthesized through high-temperature calcination using hydratable alumina (ρ-Al_2_O_3_) as precursor with water-soluble mineralizer ammonium fluoroborate (NH_4_BF_4_). ρ-Al_2_O_3_ can undergo a hydration reaction with water to form AlO(OH) and Al(OH)_3_ intermediates, serving as an excellent precursor. With the addition of 0.1 wt% NH_4_BF_4_, the product exhibits an optimal near-spherical morphology. Excessive addition (>0.2 wt%), however, significantly promotes the transformation of α-Al_2_O_3_ from a near-spherical to a plate-like structure. Further studies reveal that the introduction of NH_4_BF_4_ not only modulates the crystal morphology but also effectively reduces the content of sodium impurities in the powder through a high-temperature volatilization mechanism, thereby enhancing the thermal conductivity of the powder. It is shown that the thermal conductivity of the micron-sized α-Al_2_O_3_/ epoxy resin composites reaches 1.329 ± 0.009 W/(m·K), which is 7.4 times that of pure epoxy resin.

## 1. Introduction

The relentless pursuit of higher integration density, miniaturization, and performance in modern electronics has elevated effective thermal management to a pivotal challenge, directly impacting the longevity and reliability of these devices [1,2]. Owing to a compelling set of properties—including superior electrical insulation, low density, and ease of fabrication—polymeric thermally conductive materials have found extensive applications in areas ranging from electronic encapsulation to thermal management systems [2,3,4,5]. A fundamental limitation of polymers, however, is their inherently low thermal conductivity (typically < 0.3 W/(m·K)), which falls short of the heat dissipation demands imposed by high-power-density electronic components. Consequently, the strategy of embedding highly thermally conductive fillers into the polymer matrix is indispensable for augmenting the overall thermal transport properties of the composite. These fillers function by creating percolating networks for heat transfer within the insulating polymer, thereby markedly boosting the composite’s effective thermal conductivity. Their type, morphology, size, and content directly determine the final material’s thermal efficiency and processability [6,7,8].

The palette of available thermally conductive fillers is diverse, encompassing several categories: metal oxides (e.g., Al_2_O_3_, MgO, and ZnO), nitrides (e.g., AlN and BN), carbon allotropes (e.g., graphene and CNTs), and metallic particles (e.g., Ag and Cu), each presenting a distinct trade-off between performance, cost, and stability [2]. Each type of material has its own advantages and disadvantages in terms of thermal conductivity, cost, processability, and stability. Among these options, aluminum oxide (alumina) has emerged as the predominant filler for commercial applications, particularly in electronics, a status owed to its balanced combination of high thermal performance, electrical insulation, and low cost.

α-Al_2_O_3_ is widely valued in critical fields such as refractory materials, electronic packaging, and aerospace due to its exceptional chemical stability (including high corrosion resistance and hardness) and prominent thermophysical properties (such as high melting point and thermal conductivity) [9,10,11,12,13,14]. In α-Al_2_O_3_/polymer thermally conductive composites, heat transfer occurs primarily through phonon-mediated mechanisms [15]. Compared to fillers of other morphologies, particles with regular and near-spherical shapes exhibit a smaller specific surface area at the same particle size. This property reduces interparticle contact points and weakens van der Waals forces, thereby effectively suppressing agglomeration and facilitating improved flow and dispersion within the polymer matrix. Furthermore, smooth and geometrically simple interfaces help minimize phonon scattering, significantly reducing interfacial thermal resistance. Owing to their high intrinsic thermal conductivity, low interfacial thermal resistance, and excellent flow and packing characteristics, spherical or near-spherical α-Al_2_O_3_ particles have become a preferred choice as high-performance thermal fillers in electronic devices [16,17,18,19].

The common synthesis methods for spherical α-Al_2_O_3_ primarily include the sol–gel method, homogeneous precipitation, hydrothermal synthesis, and molten jet spraying. Both the sol–gel and homogeneous precipitation methods enable the preparation of α-Al_2_O_3_ powders with controllable particle size, diverse morphology, and high purity at relatively low temperatures by regulating the particle morphology, size, and specific surface area of aluminum-containing precursors [11,20,21,22]. However, the sol–gel method involves high raw material costs, complex processes, and often employs large amounts of organic solvents in the reaction system. Although homogeneous precipitation offers cost advantages, it may generate harmful sulfur-containing by-products during the calcination process. The hydrothermal method can directly synthesize spherical or near-spherical α-Al_2_O_3_ at relatively low temperatures (around 450 °C) [23,24]. Nevertheless, this method requires sophisticated equipment, leading to significantly increased capital investment and unit production costs, and demands extremely precise control over reaction parameters such as pH and filling degree. Molten jet spraying typically uses high-purity alumina as a raw material, producing spherical products through high-temperature melting and spray granulation [25,26,27]. As a result, individual spherical α-Al_2_O_3_ microbeads are usually polycrystalline and often contain intrinsic defects such as pores and vacancies within the crystal structure. Moreover, due to the extremely high melting point of industrial alumina (>2050 °C), this process imposes stringent requirements on the high-temperature resistance of equipment, resulting in high capital and operating costs. In contrast, near-spherical alumina can be obtained via specific calcination processes and the use of calcination aids [12,13], resulting in single-crystal products with uniform grain size and higher α-phase content. Their regular surface morphology and good fluidity help reduce agglomeration within the matrix and improve dispersion uniformity during processing. By optimizing crystal structure and particle size distribution, high packing density (up to 70 vol% or more) can be achieved, strengthening the thermal conduction pathways and significantly enhancing the thermal conductivity of the composite, thereby meeting the heat dissipation demands of most electronic devices.

ρ-Al_2_O_3_ is a transitional alumina phase with high reactivity, capable of undergoing a hydration reaction with water to form a high-strength aluminum hydroxide gel. It is commonly used as a binder in refractories and other related fields [28,29,30]. The hydration products, AlO(OH) and Al(OH)_3_ [31], can serve as precursors for the preparation of alumina powders via solid-phase calcination. NH_4_BF_4_ is a chemical compound widely used in analytical reagents, insecticides, and as a catalyst for resin finishing in the textile industry. In recent years, it has been exploited by researchers for modulating the crystal morphology of alumina [13]. The aqueous gel formed by the hydration of ρ-Al_2_O_3_ can dissolve most mineralizers, enabling their uniform dispersion within the hydration products. This enhances the interaction between the mineralizer and the hydration products of ρ-Al_2_O_3_, thereby achieving the goal of homogeneous primary crystal formation. This hydration characteristic of ρ-Al_2_O_3_ is highly advantageous for implementing strategies to tailor the properties of α-alumina powders, leading to the production of high-quality powders with excellent morphology and performance. NH_4_BF_4_ is highly soluble in water, which facilitates better mixing with ρ-Al_2_O_3_. Therefore, based on the report by Zhenhao Yang et al. [13], this study used ρ-Al_2_O_3_ as the raw material, optimized the selection of precursors, and introduced NH_4_BF_4_ as a mineralizer to prepare micron-sized spherical α-Al_2_O_3_ powders. The effects of the amount of NH_4_BF_4_ addition on the morphology of α-Al_2_O_3_ powders and the reduction of sodium impurity content were systematically investigated, and the underlying regulation mechanisms were further analyzed.

## 2. Experimental

### 2.1. Preparation of Near-Spherical Alumina Powder of Micron Size

60 g of ρ-Al_2_O_3_ powder (supplied by the Aluminum Corporation of China, Chalco, Zhengzhou, China) and 140 g of deionized water were placed in a 500 mL zirconia ball milling jar, giving a solid-to-liquid mass ratio of 3:7. The NH_4_BF_4_ mineralizer, which was obtained from Macklin Biochemical Co., Ltd. in Shanghai, China, was added in a predetermined proportion based on the mass fraction of ρ-Al_2_O_3_. Then, 120 g of zirconia grinding balls (5 mm in diameter, at a ball-to-powder mass ratio of 2:1) were added to the jar. The mixture was mechanically blended using a KQM-Z/B planetary ball mill (supplied by Xianyang JinHong General Machinery Co., Ltd., Xianyang, China) at 300 r/min for 3 h to ensure a thorough and uniform dispersion of the mineralizer and the precursor.

The ball-milled slurry was transferred into a cylindrical silicone mold (Φ50 mm × 50 mm), covered with a sealing film, and subjected to hydration treatment at 60 °C for 24 h in a constant-temperature drying oven (HWS-150; Shaoxing Huda Machinery Equipment Manufacturing Co., Ltd., Shaoxing, China). After removing the sealing film, drying was continued at the same temperature for another 24 h to obtain a free-standing gel-derived green body. The dried green body was loaded into a 100 mL arc-shaped corundum crucible. The crucible was then covered with its matching arc-shaped lid to ensure a partially sealed environment. It was subsequently transferred to a chamber furnace (KAL-1700X; Hefei Kjing Materials Technology Co., Ltd., Hefei, China) for stepwise calcination: the temperature was first raised to 1100 °C at 5 °C/min, then increased to the target temperature (1300 °C) at 3 °C/min (The heating rate was set to the maximum achievable rate of the chamber furnace within the corresponding temperature range.), and finally held at that temperature for 1–4 h. Micron-scale near-spherical α-Al_2_O_3_ powder was obtained after furnace cooling. The cooling process from 1300 °C to 500 °C was controlled by the furnace, with the cooling rate set equal to the heating rate. Upon reaching 500 °C, the program was halted, and the sample was allowed to cool naturally.

### 2.2. Preparation of α-Al_2_O_3_/EP Thermally Conductive Composites

Seventy weight percent (70 wt%) of self-synthesized micron-sized near-spherical α-Al_2_O_3_ powder was incorporated into the EP gel (CYDF-170; viscosity: 8000–12,000 mPa·s at 25 °C; thermal conductivity: 0.18 W·m^-1^·K^-1^; supplied by Jiangyin Chengyu New Material Technology Co., Ltd., Jiangyin, China)and mixed using a three-roll mill (S100; Shanghai Root Mechanical and Electrical Equipment Co., Ltd., Shanghai, China). All mixing was performed at room temperature. The equipment parameters were set as follows: slow roller speed at 30 r/min, middle roller speed at 70 r/min, and fast roller speed at 150 r/min. The mixture was processed continuously for three cycles, with each cycle lasting 2 h. Afterwards, a curing agent (methyltetrahydrophthalic anhydride supplied by Aladdin Biochemical Technology Co., Ltd., Shanghai, China) was added at a mass ratio of 5:6 and thoroughly mixed by mechanical stirring.

The uniformly mixed material was then degassed for 1 h in a vacuum environment to remove residual internal bubbles and ensure the density of the subsequently molded samples. Following degassing, the α-Al_2_O_3_/EP mixture was slowly poured into a mold pre-treated with a release agent. The mold was transferred to a vacuum oven (DZF-6050; Shanghai Yiheng Technical Co., Ltd., Shanghai, China) and cured at 160 °C for 10 h. After curing, the mold was allowed to cool naturally to room temperature before demolding.

### 2.3. Characterizations

Phase analysis of the powder samples was conducted using an X-ray diffractometer (XRD, Bruker D8 DISCOVER A25, Bruker AXS GmbH, Karlsruhe, Germany) with a scanning range of 10° to 90° and a scanning rate of 5°/min. The microscopic morphology of the powder was observed using a field-emission scanning electron microscope (SEM, JEOL SM-76190F, JEOL Ltd., Tokyo, Japan), with a magnification voltage of 10 kV, and the magnification levels are 1000, 3000, and 5000. The crystal structure of the powder was characterized by transmission electron microscopy (TEM, Thermo Scientific Talos F200X, Thermo Fisher Scientific, Waltham, MA, USA) and selected area electron diffraction (SAED). The phase transition behavior of alumina was analyzed using a simultaneous thermal analyzer (STA, Netzsch STA 449 F5, Netzsch-Gerätebau GmbH, Selb, Germany). Powder samples were tested in the temperature range of 30 °C to 1300 °C at a heating rate of 20 °C/min to obtain TG-DSC curves. Grain size distribution was analyzed using a laser diffraction particle size analyzer (LS-POP (9); OMEC Instruments Co., Ltd., Zhuhai, China) and Nanomeasurer 1.2 software (Jade Biomedical Technology Co., Ltd., Shanghai, China). The sodium (Na) content in the sample was determined by inductively coupled plasma optical emission spectrometry (ICP-OES). The thermal conductivity of the composite in the through-plane direction was measured using the laser flash method (LFA 467, NETZSCH-Gerätebau GmbH, Selb, Germany).

## 3. Results and Discussion

**Figure 1:**
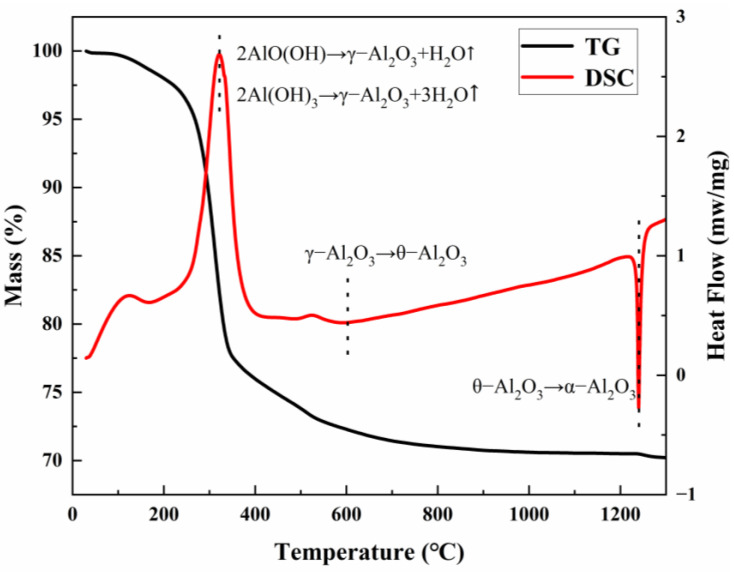
TG-DSC curve of ρ-Al_2_O_3_ after hydration for 24 h.

Figure 1 shows the simultaneous thermal analysis (TG-DSC) curve of the product of ρ-Al_2_O_3_ hydration for 24 h. Two characteristic endothermic peaks and two exothermic phase transition peaks can be observed in the DSC curve. The endothermic peak observed at 121 °C is attributed to the desorption of physically adsorbed water, while the endothermic effects at 320 °C and 550 °C correspond to the stepwise dehydration of Al(OH)_3_ (bayerite) and AlO(OH) (boehmite), respectively, leading to the formation of metastable γ-Al_2_O_3_. The corresponding TG curve showed continuous weight loss behavior in this temperature range, and the total weight loss rate reached 25%, which was highly consistent with the theoretical dehydration (adsorbed water + structural hydroxyl group removal). The exothermic peak of the DSC curve at 610 °C corresponds to the enthalpy of phase change released by lattice reconstruction during the phase transition of γ-Al_2_O_3_ →θ-Al_2_O_3_. The strong exothermic peak at 1240 °C is attributed to the irreversible phase transition of θ-Al_2_O_3_→α-Al_2_O_3_. It is worth noting that the α phase generation peak is fully present before 1300 °C, indicating that the phase transition process can be fully completed at this temperature. Based on this thermodynamic analysis, 1300 °C was selected as the optimal calcination temperature to ensure the high-purity conversion of the α phase.

**Figure 2:**
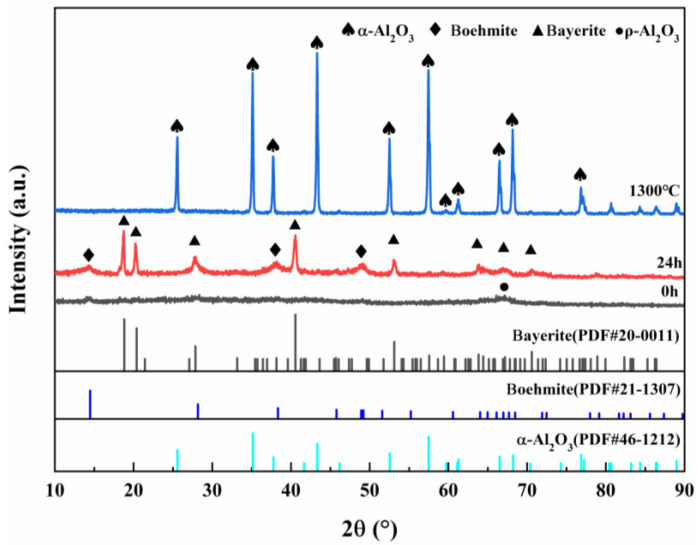
XRD patterns of ρ-Al_2_O_3_ feedstock, hydrated 24 h products, and products calcined at 1300 °C for 2 h.

Figure 2 shows the XRD plots of ρ-Al_2_O_3_ feedstock, hydrated 24 h products, and products calcined at 1300 °C for 2 h, and the diffraction peaks of ρ-Al_2_O_3_ can be observed as amorphous diffuse peaks, which are consistent with the results reported in the literature [31]. After 24 h of ρ-Al_2_O_3_ hydration, the diffraction peak of the product conformed to the PDF cards of Boehmite and Bayerite, indicating that the hydration product was composed of Boehmite and Bayerite. The diffraction peak of the hydration product was consistent with the PDF card of α-Al_2_O_3_, and there was no diffraction peak of θ-Al_2_O_3_, indicating that the pure phase α-Al_2_O_3_ could be obtained after calcination at 1300 °C for 2 h.

**Figure 3:**
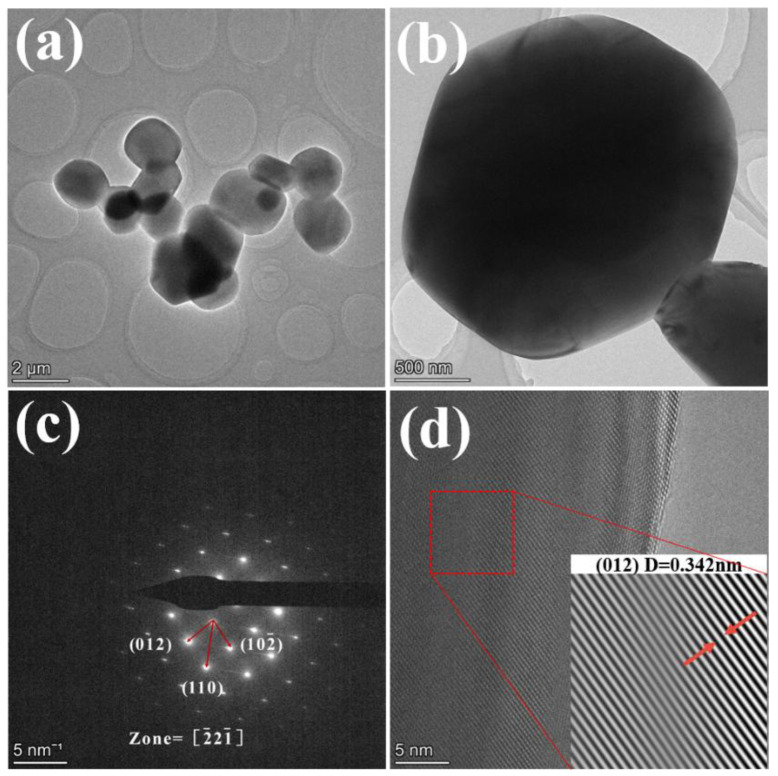
TEM results of the sample with 0.1% NH_4_BF_4_ addition after calcination at 1300 °C for 2 h (**a**,**b**) are TEM images at different magnifications (**c**) SAED (**d**) HRTEM.

Figure 3 shows the TEM test results of the sample with 0.1% NH_4_BF_4_ addition after calcination at 1300 °C for 2 h. From Figure 3a,b, it can be seen that the sample particles exhibit a near-spherical topography with a particle size between 1 μm and 2 μm. The results are shown in Figure 3c, indicating that the sample has a tripartite phase structure, and the results of the crystal plane index identification corresponding to the diffraction spot are consistent with the tripartite phase. Figure 3d shows the high-resolution HRTEM results, the Fourier transform and the reverse Fourier transform are performed on the red frame area to obtain the lattice fringes of the sample, and the lattice fringe spacing measured at the arrow is 0.342 nm, which is consistent with the spacing of the (012) crystal plane, indicating that the substance is α-Al_2_O_3_.

**Figure 4:**
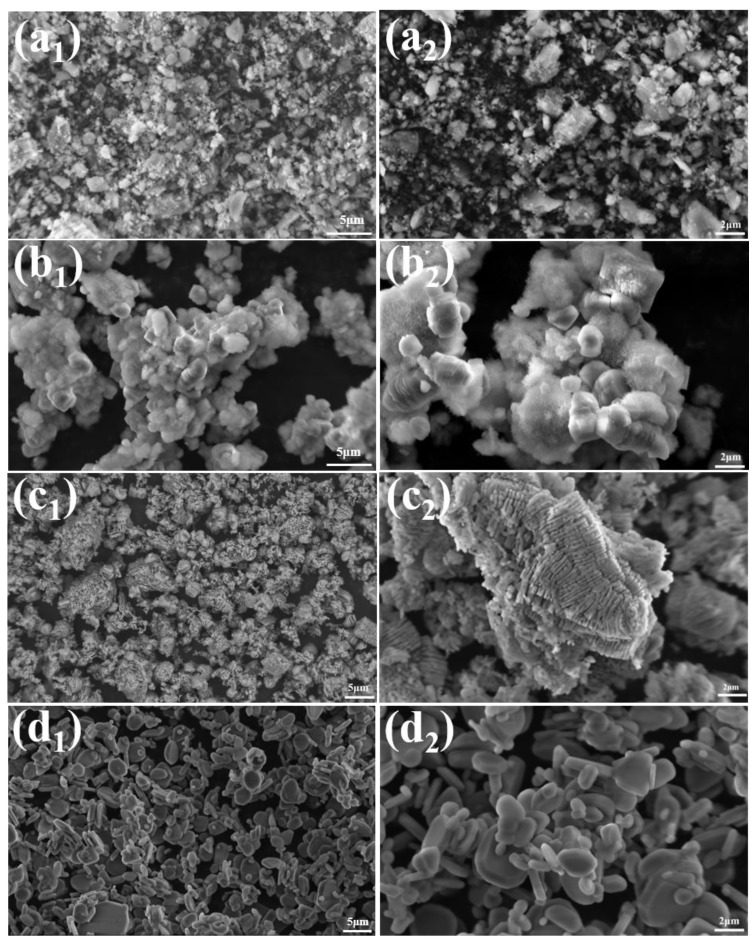
SEM images of (**a_1_**,**a_2_**) ρ-Al_2_O_3,_ (**b_1_**,**b_2_**) hydration 24 h products, (**c_1_**,**c_2_**) hydration product was calcined at 1300 °C for 2 h without NH_4_BF_4_, (**d_1_**,**d_2_**) hydration product was calcined at 1300 °C for 2 h with 0.4% NH_4_BF_4_.

Figure 4 shows the SEM images of ρ-Al_2_O_3_, as observed in Figure 4a_1_,a_2_, the raw material ρ-Al_2_O_3_ exhibits an irregular morphology. The microstructure of the sample after 24 h of hydration of ρ-Al_2_O_3_ is shown in Figure 4b_1_,b_2_, which is primarily composed of flocculent and particulate matter. The study conducted by R. Salomão et al. [32] provided a detailed investigation into the hydration of ρ-Al_2_O_3_, confirming that the flocculent material is AlO(OH) and the particulate matter is Al(OH)_3_. The hydration product was calcined at 1300 °C for 2 h, and the sample without NH_4_BF_4_ was transformed into adherent worm-like α-Al_2_O_3_. This is because, without the addition of any mineralizing agent, the mass transfer mode between alumina is solid-phase mass transfer, and the parts that come into contact with each other will form interlocking structures, which in turn will form an adhesion dumbbell-shaped morphology [12]. The samples added with 0.4% NH_4_BF_4_ were transformed into flakes and near-spherical α-Al_2_O_3_, and the results showed that NH_4_BF_4_ could regulate the morphology of alumina.

**Figure 5:**
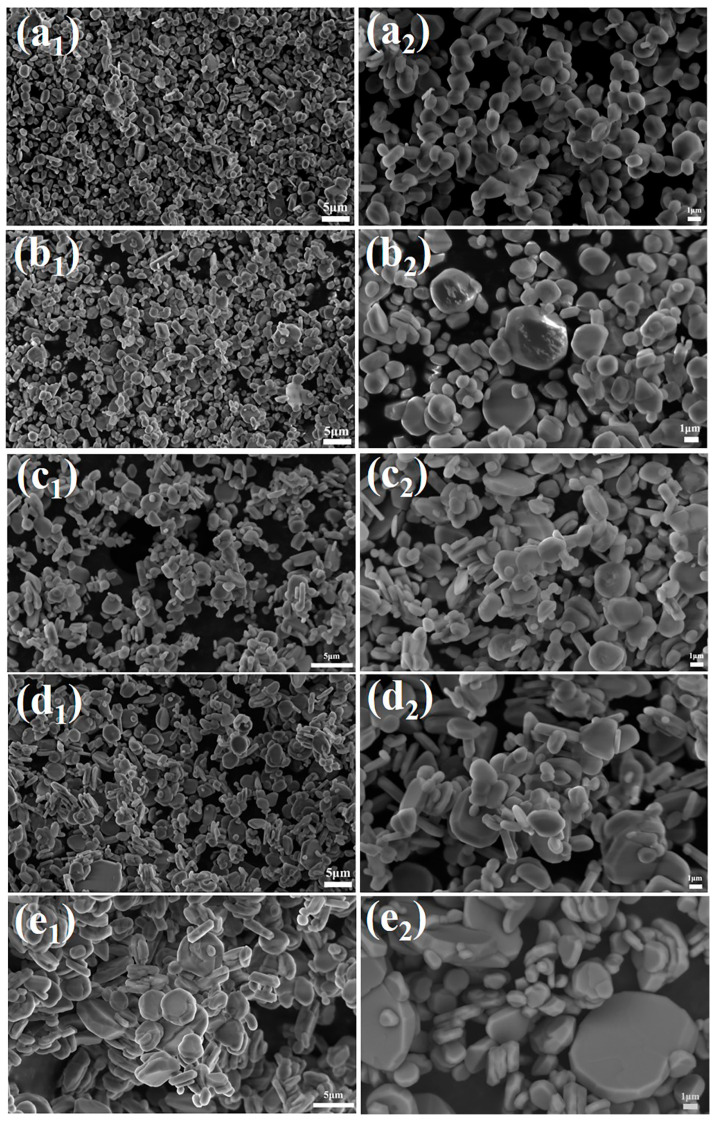
SEM images of calcination products of samples with different dosages calcined at 1300 °C for 2 h (**a_1_**,**a_2_**) 0.1%, (**b_1_**,**b_2_**) 0.2%, (**c_1_**,**c_2_**) 0.3%, (**d_1_**,**d_2_**) 0.4%, (**e_1_**,**e_2_**) 0.5%.

Figure 5 shows the SEM images of the calcination products of samples with different dosages calcined at 1300 °C for 2 h. As can be seen from Figure 5, 0.1% NH_4_BF_4_ was added, the α-Al_2_O_3_ grains in the sample exhibited a predominantly near-spherical morphology with high sphericity, good dispersion among grains, and no significant agglomeration. As the amount of NH_4_BF_4_ increased, the near-spherical α-Al_2_O_3_ grains became progressively flattened with a non-uniform size distribution. When the addition amount was further increased to 0.5%, the morphology of the α-Al_2_O_3_ grains almost entirely transitioned to a plate-like shape. These results indicate that under the experimental conditions applied in this study, 0.1% NH_4_BF_4_ is the optimal addition amount for obtaining near-spherical α-Al_2_O_3_. Excessive addition disrupts the coordination balance during crystal growth, leading to a transition in grain morphology from near-spherical to plate-like.

**Figure 6:**
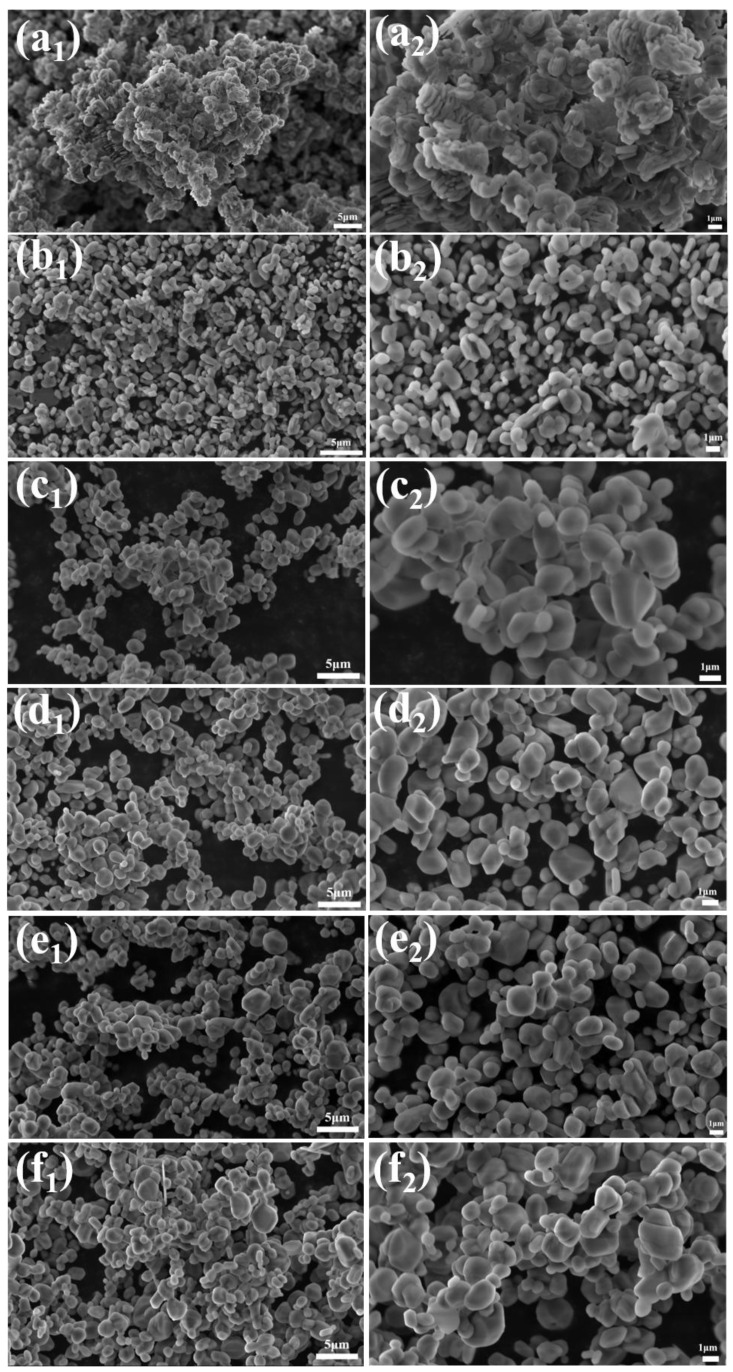
SEM images of calcination products of samples with different dosages calcined at 1300 °C for 2 h (**a_1_**,**a_2_**) 0.04%, (**b_1_**,**b_2_**) 0.06%, (**c_1_**,**c_2_**) 0.08%, (**d_1_**,**d_2_**) 0.12%, (**e_1_**,**e_2_**) 0.14%, and (**f_1_**,**f_2_**) 0.16%.

Based on the results from the previous set of experiments, a further experiment was designed to finely adjust the amount of NH_4_BF_4_ addition (with fine-tuning in the range of 0.04–0.16%). The morphological evolution was analyzed using its SEM images (as shown in Figure 6), revealing the dynamic regulation mechanism of the α-Al_2_O_3_ grain morphology.

In the sample with 0.04% NH_4_BF_4_ addition, the α-Al_2_O_3_ grains exhibited a highly agglomerated vermicular structure, with lengths ranging from 1 to 4 μm. The grains were interconnected, forming network-like stacked aggregates. This morphology results from the weak regulating effect of BF_4_^−^ ions on crystal facet growth when the NH_4_BF_4_ concentration is insufficient, leading to preferential growth along the (104) crystal plane and mutual adhesion among crystals.

As the amount of NH_4_BF_4_ addition increases, the vermicular α-Al_2_O_3_ grains gradually disassociate, transforming first into an elliptical shape and then into a near-spherical shape. From Figure 6, it can be observed that in samples with more than 0.1% NH_4_BF_4_ addition, the α-Al_2_O_3_ grains exhibit a flattened morphology with irregular sizes. Therefore, 0.1% NH_4_BF_4_ is identified as the optimal addition amount.

**Figure 7:**
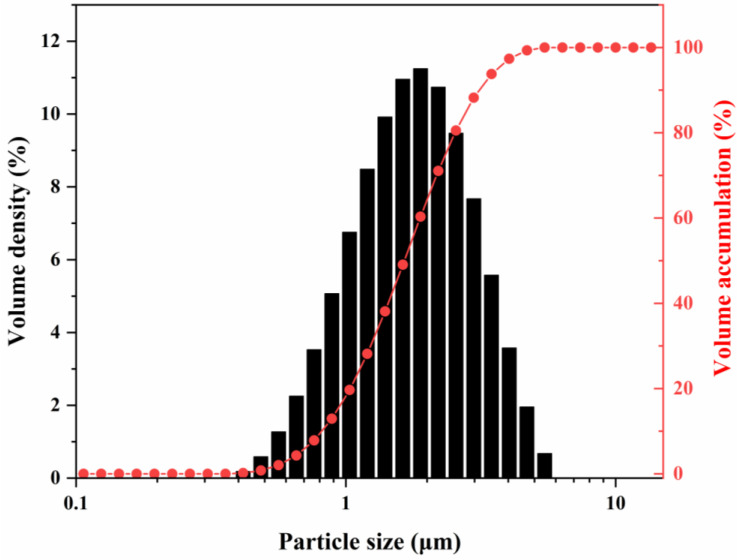
Particle size distribution of alumina powders calcined at 1300 °C for 2 h with 0.1% NH_4_BF_4_.

Figure 7 shows the particle size distribution of the calcination product obtained by calcination at 1300 °C for 2 h with 0.1% NH_4_BF_4_. Particle size analysis revealed that the D10, D50, and D90 values were 0.81 μm, 1.65 μm, and 3.11 μm, respectively, with a span of 1.39, indicating a moderate dispersion of the particles. Micron-sized particles accounted for 83% of the total. This multi-scale particle size distribution facilitates tighter packing of the fillers within the matrix—wherein larger particles form a primary framework while finer particles fill the interparticle voids—thereby enhancing phonon transport efficiency and contributing to improved thermal conductivity of the composite material. This mechanism is supported by the study of Wang et al. [33], in which an optimized alumina filler system (60 wt% loading, with a particle size ratio of 5.8 μm:2.6 μm:0.5 μm = 7:2:1) exhibited a 23–32% increase in thermal conductivity compared to systems using a single particle size.

**Figure 8:**
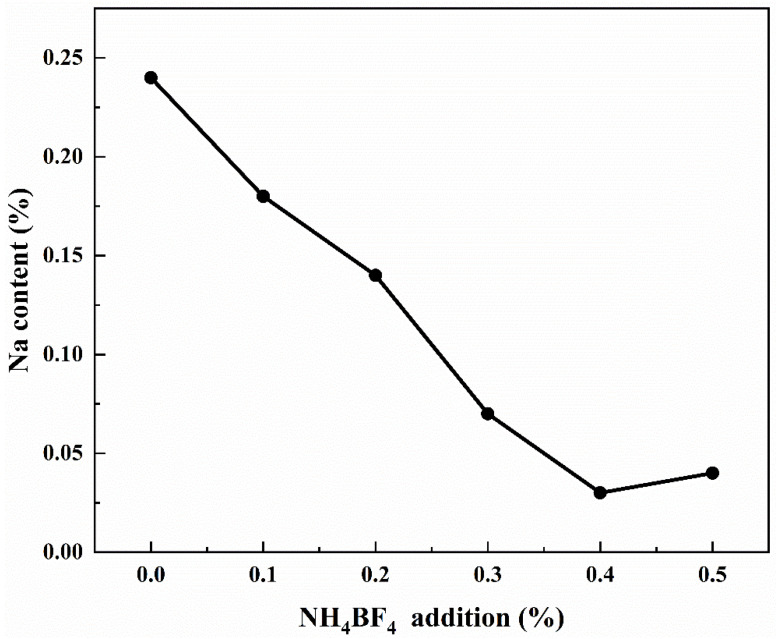
The Na content of samples calcined at 1300 °C for 2 h with NH_4_BF_4_ addition.

Since the vast majority of bauxite worldwide is processed using the Bayer process—an alkaline method that utilizes a significant amount of sodium hydroxide solution—industrial-grade Al(OH)_3_, Al_2_O_3_, and other aluminum precursors produced via this method typically contain certain amounts of residual Na impurities [34]. Figure 8 shows the test results of Na content of samples calcined at 1300 °C for 2 h with NH_4_BF_4_ addition, and it can be observed from Figure 8 that the addition of NH_4_BF_4_ can reduce the content of impurity Na in the calcination products, and the impurity Na content in the sample decreases with the increase of NH_4_BF_4_ addition, from the initial 0.24% to 0.03%, as the high-temperature decomposition of NH_4_BF_4_ will form intermediate products containing element B and F. The intermediate product containing elements B and F reacts with Na_2_O to form NaBO_2_ and NaF, which volatilize at high temperatures, thereby reducing the content of the impurity Na [12].

**Figure 9:**
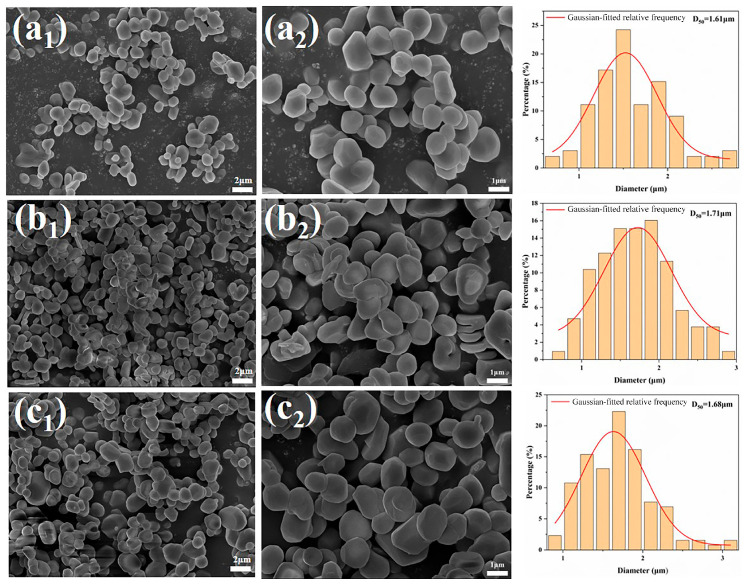
The SEM images of the samples calcined at 1300 °C for different durations with 0.1% NH_4_BF_4_ (**a_1_**,**a_2_**) 1 h, (**b_1_**,**b_2_**) 3 h, (**c_1_**,**c_2_**) 4 h.

Generally speaking, when α-Al_2_O_3_ is prepared by high-temperature calcination, the grain size can be increased by extending the holding time to promote the one-time grain growth. Figure 9 shows the SEM images of the samples with 0.1% NH_4_BF_4_calcined at 1300 °C for different durations. As can be observed from Figure 9, there is basically no difference in morphology and particle size between the samples obtained at 1300 °C for 1 h and those kept at 1300 °C for 3 h. The results showed that the near-spherical α-Al_2_O_3_ powder prepared by adding NH_4_BF_4_ was not sensitive to the holding time and had excellent thermal stability.

NH_4_BF_4_ undergoes thermal decomposition at high temperatures, forming gaseous compounds such as HF, NH_3_, and BF_3_. The fluorine-containing products further generate gaseous intermediate AlOF during the high-temperature calcination process. The reaction equation is as follows:

(1) $${NH}_{4}{BF}_{4}\to {NH}_{3}+HF+{BF}_{3}$$

(2) $${BF}_{3}+3H_{2}O\to H_{3}{BO}_{3}+3HF$$

(3) $$2{BF}_{3}+{Al}_{2}O_{3}\to 2AlF_{3}+B_{2}O_{3}$$

(4) $$6HF+{Al}_{2}O_{3}\to 2AlF_{3}+3H_{2}O$$

(5) $$AlF_{3}+H_{2}O\to AlOF+2HF$$

(6) $$3AlOF\to {Al}_{2}O_{3}+AlF_{3}$$

(7) $$2AlOF+H_{2}O\to {Al}_{2}O_{3}+2HF$$

**Figure 10:**
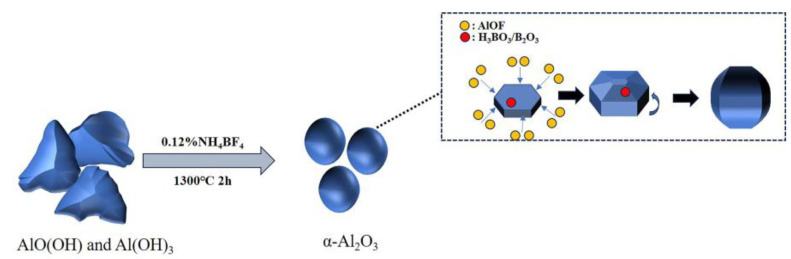
Schematic diagram of the mechanism of NH_4_BF_4_ action.

Figure 10 illustrates the schematic diagram of the mechanism of NH_4_BF_4_ action. Due to the fact that the (0001) crystal plane and its symmetrical equivalents in α-Al_2_O_3_ crystals are closely packed planes with relatively low surface free energy and poor adsorption capacity compared to the other six crystal planes, AlOF is preferentially adsorbed onto the other six planes, leading to plate-like growth [12]. However, when H_3_BO_3_ is introduced into the system, it may interact with surface hydroxyl groups on the crystals, reducing the growth activation energy and accelerating three-dimensional grain growth. On the other hand, borate ions generated from the decomposition or hydrolysis of H_3_BO_3_ at high temperatures may incorporate into the crystal lattice or adsorb at grain boundaries, altering the local ionic environment and further promoting grain growth [35]. As a result, the addition of H_3_BO_3_ may cause plate-like grains to thicken and increase in size, driving a morphological transition toward a spherical-like shape.

The mechanism by which NH_4_BF_4_ modulates morphology may involve its initial decomposition into gaseous compounds at high temperatures, which subsequently react with water and Al_2_O_3_ to form AlF_3_ and H_3_BO_3_. Here, H_3_BO_3_ promotes lateral growth and increases particle size, while AlF_3_ may inhibit the growth of specific crystal planes or alter interfacial energy. Under the synergistic influence of F and B elements, the crystal growth direction is altered, ultimately resulting in the formation of spherical-like alumina particles with increased size.

However, the influence of NH_4_BF_4_ on morphology and size is highly dependent on its dosage. When the amount added is low, the insufficient release of gaseous species and low concentration of H_3_BO_3_ result in agglomerated worm-like particles with limited size increase. When excessive amounts are added, the increased concentration of AlF_3_ may suppress growth in the thickness direction, leading to a plate-like morphology. Only at an optimal dosage can the synergistic effects of H_3_BO_3_ and AlF_3_ promote growth to a suitable size and regulate the morphology toward a spherical-like shape.

Micron-sized α-Al_2_O_3_ powder, obtained by adding 0.1% NH_4_BF_4_ followed by calcination at 1300 °C, was used as the thermal conductive filler to prepare composites with epoxy resin (EP). The thermal conductivity test results of the micron-sized α-Al_2_O_3_/EP composites are summarized in Table 1. Four samples from the same batch were tested, and the composite sample exhibited a thermal conductivity of 1.329 W/(m·K). Compared with recently reported polymer composites filled with α-Al_2_O_3_, even at higher filler loadings of conventional micron-sized α-Al_2_O_3_, the thermal conductivity of such composites mostly falls within the range of 0.5–0.9 W/(m·K) [36], which is significantly lower than the value achieved in this study. This result highlights the remarkable advantage of the filler prepared in this work in enhancing the thermal conductivity of composites.

## 4. Conclusions

In this study, micron-scale near-spherical α-Al_2_O_3_ powder was synthesized via solid-phase sintering using ρ-Al_2_O_3_ as the raw material and NH_4_BF_4_ as a mineralizer. The effects of NH_4_BF_4_ addition, calcination temperature, and holding time on the morphology and particle size of the resulting α-Al_2_O_3_ were systematically investigated. The prepared powder exhibited a median particle size (D_50_) of 1.65 μm, and the mechanistic role of NH_4_BF_4_ appears to be as follows, based on the obtained results:

(1) The addition of NH_4_BF_4_ effectively modulates the crystal morphology of α-Al_2_O_3_, with a pronounced correlation to the amount added. The underlying mechanism involves the thermal decomposition of NH_4_BF_4_ into fluorine-containing (e.g., HF and AlF_3_) and boron-containing (e.g., H_3_BO_3_) intermediates. Fluorine and boron act synergistically to alter the surface energy distribution at the crystal growth interface, thereby suppressing anisotropic growth and promoting equiaxial development, leading to the formation of a near-spherical morphology. Experimental results show that with 0.1% NH_4_BF_4_ addition, near-spherical α-Al_2_O_3_ powder with D_50_ = 1.65 μm was obtained. Increasing the addition to 0.5% resulted in a loss of morphological control due to an imbalance in the fluorine-to-boron ratio, yielding a mixture of near-spherical and plate-like α-Al_2_O_3_. In contrast, reducing the addition to 0.04% provided insufficient mineralizer concentration, resulting in entirely agglomerated vermicular α-Al_2_O_3_ without effective morphology regulation.
(2) NH_4_BF_4_ serves as an efficient mineralizer that facilitates the formation of α-Al_2_O_3_. Under these conditions, the holding time has minimal influence on the final crystal morphology and particle size. Experiments confirmed that within a 1–4 h holding period, the morphology and size of the α-Al_2_O_3_ crystals remained largely unchanged, with no significant agglomeration or abnormal grain growth—markedly superior to the control group without NH_4_BF_4_. This behavior is attributed to gaseous products from NH_4_BF_4_ decomposition, which regulate grain-boundary diffusion by reducing interfacial energy and suppressing Ostwald ripening, thereby decoupling grain growth rate from holding time.
(3) By introducing 0.1% NH_4_BF_4_ and calcining at 1300 °C, near-spherical α-Al_2_O_3_ powder with D_50_= 1.65 μm was successfully prepared. The composite gel formed with epoxy (EP) exhibited a thermal conductivity of 1.329 ± 0.009 W/(m·K). These findings provide a feasible strategy for the industrial production of micron-scale near-spherical α-Al_2_O_3_ powder.

Although this study optimized the selection of precursors and utilized NH_4_BF_4_ as a mineralizer to modulate morphology, along with an in-depth analysis of the underlying mechanism, conclusive evidence has not yet been established due to experimental constraints. Furthermore, only the thermal conductivity of the prepared composite material was characterized, and its practical applications still require further validation. Therefore, we propose that subsequent research should focus on verifying the proposed mechanism and exploring specific applications of the composite material.

## Figures and Tables

**Table 1:** Test results of thermal conductivity for micron-sized α-Al_2_O_3_/EP composites.

Whiteness %	Oil Absorption 10 g/100 g	Moisture Content	PH 10 g/100 g	Electrical Conductivity μs/cm	Thermal Conductivity w/(m·k)	Extrusion g/min
99.4	53.97	0.29	9.11	94.80	1.329 ± 0.009	32.76

## Data Availability

The original contributions presented in this study are included in the article. Further inquiries can be directed to the corresponding authors.
